# Development of rubber‐enriched dandelion varieties by metabolic engineering of the inulin pathway

**DOI:** 10.1111/pbi.12672

**Published:** 2017-02-09

**Authors:** Anna Stolze, Alan Wanke, Nicole van Deenen, Roland Geyer, Dirk Prüfer, Christian Schulze Gronover

**Affiliations:** ^1^ Institute of Plant Biology and Biotechnology University of Muenster Muenster Germany; ^2^ numares AG Regensburg Germany; ^3^ Fraunhofer Institute for Molecular Biology and Applied Ecology (IME) Muenster Germany

**Keywords:** *Taraxacum koksaghyz*, natural rubber, triterpene, inulin, fructosyltransferase, fructan 1‐exohydrolase

## Abstract

Natural rubber (NR) is an important raw material for a large number of industrial products. The primary source of NR is the rubber tree *Hevea brasiliensis*, but increased worldwide demand means that alternative sustainable sources are urgently required. The Russian dandelion (*Taraxacum koksaghyz* Rodin) is such an alternative because large amounts of NR are produced in its root system. However, rubber biosynthesis must be improved to develop *T. koksaghyz* into a commercially feasible crop. In addition to NR,* T. koksaghyz* also produces large amounts of the reserve carbohydrate inulin, which is stored in parenchymal root cell vacuoles near the phloem, adjacent to apoplastically separated laticifers. In contrast to NR, which accumulates throughout the year even during dormancy, inulin is synthesized during the summer and is degraded from the autumn onwards when root tissues undergo a sink‐to‐source transition. We carried out a comprehensive analysis of inulin and NR metabolism in *T. koksaghyz* and its close relative *T. brevicorniculatum* and functionally characterized the key enzyme fructan 1‐exohydrolase (1‐FEH), which catalyses the degradation of inulin to fructose and sucrose. The constitutive overexpression of *Tk1‐FEH* almost doubled the rubber content in the roots of two dandelion species without any trade‐offs in terms of plant fitness. To our knowledge, this is the first study showing that energy supplied by the reserve carbohydrate inulin can be used to promote the synthesis of NR in dandelions, providing a basis for the breeding of rubber‐enriched varieties for industrial rubber production.

## Introduction

Natural rubber is a unique and economically important biopolymer mainly produced by the rubber tree *Hevea brasiliensis* (Schulze Gronover *et al*., 2011). However, the increasing demand for NR (>12.1 million tons in 2014) and adverse influences, such as climate change, vulnerable *H. brasiliensis* monocultures and their replacement by more profitable oil palms, have encouraged the search for alternative NR‐producing plants (Arias *et al*., 2016). The annual or perennial Russian dandelion (*Taraxacum koksaghyz*) synthesizes high molecular mass poly(*cis*‐1,4‐isoprene) in specialized latex‐producing tubular cells known as laticifers and therefore offers an alternative source of NR (Epping *et al*., 2015). Although laticifers are also found in pedicels and leaves, NR is mainly synthesized in the *T. koksaghyz* root system.

**Figure 1:**
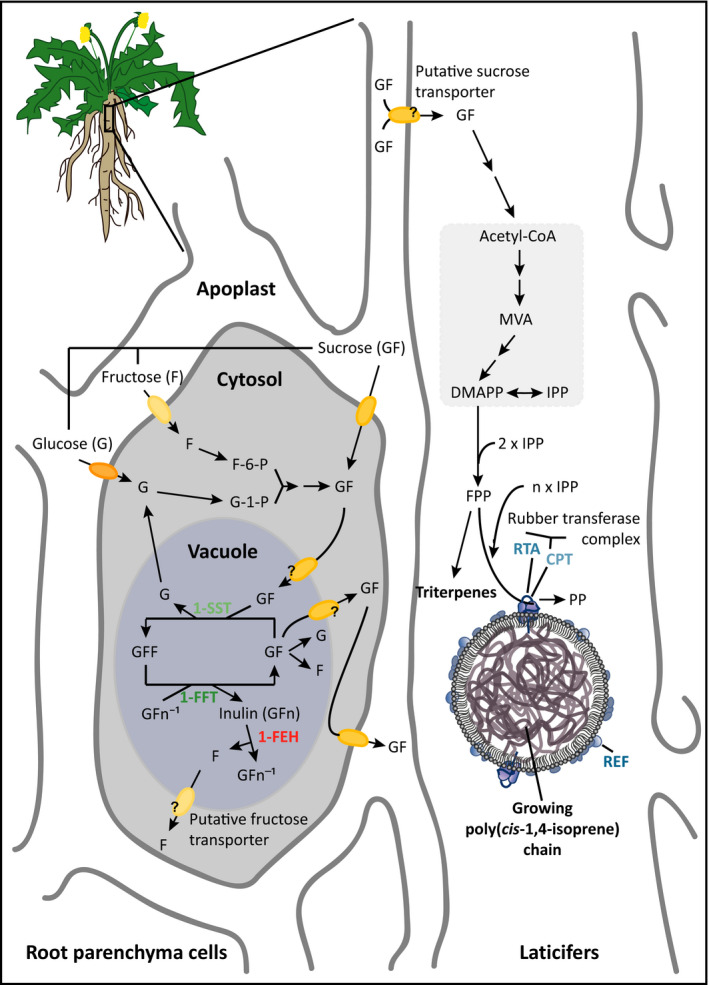
Putative model of the inulin and isoprenoid metabolic network in dandelion roots. Sucrose, either transferred from the apoplast or synthesized from glucose and fructose in the cytosol of the parenchymal root cells, is transported into the vacuole and used as a substrate for inulin biosynthesis. The degradation of inulin by 1‐FEH produces free sucrose and fructose. Sucrose is actively transported through the cytoplasm and apoplast into the laticifers and is used as a precursor via the mevalonate pathway for the synthesis of isopentenyl pyrophosphate (IPP), the basic building block of isoprenoids such as triterpenes and poly(*cis*‐1,4‐isoprene). F‐6‐P, fructose‐6‐phosphate; G‐1‐P, glucose‐1‐phosphate; GFF, 1‐kestose; MVA, mevalonic acid; DMAPP, dimethylallyl pyrophosphate; FPP, farnesyl pyrophosphate.

The basic building block of NR is isopentenyl pyrophosphate (IPP), which is thought to be produced mainly via the cytosolic mevalonic acid (MVA) pathway (van Deenen *et al*., 2012). The same pathway also provides IPP for the synthesis of various isoprenoid end products, including sterols and pentacyclic triterpenes (Figure 1) that fulfil important roles in membrane fluidity, development and resistance against herbivores (Huber *et al*., 2015; Schaller, 2003). In *H. brasiliensis*, sucrose is thought to be the exclusive precursor of IPP and is actively translocated into the laticifers by sucrose transporters (Dusotoit‐Coucaud *et al*., 2009, 2010).

In *T. brevicorniculatum*, an apomictic close relative of *T. koksaghyz* (which reproduces sexually and undergoes obligatory outcrossing), NR is synthesized on the surface of rubber particles, which are stabilized by auxiliary proteins such as the rubber elongation factor (REF) (Laibach *et al*., 2015). The elongation of the IPP chain is catalysed by a rubber *cis*‐prenyltransferase (CPT) complex whose activity is enhanced by the presence of a rubber transferase activator (RTA) (Epping *et al*., 2015). In *T. brevicorniculatum*, NR typically represents about 0.5% of the root dry weight (DW) (Epping *et al*., 2015; Post *et al*., 2012). In contrast, *T. koksaghyz* shows considerable intraspecific genetic and phenotypic diversity, and the NR content varies between 2% and 15% DW in this species (Koroleva, 1940; Van Beilen and Poirer, 2007).

Inulin, a linear β‐(2→1)‐linked fructan, is another abundant dandelion metabolite that accumulates exclusively in the roots to levels exceeding 50% DW (Van den Ende *et al*., 2000b). Fructans are water‐soluble reserve carbohydrates that are thought to be synthesized and stored within the vacuoles in ~15% of all angiosperm species, including chicory (*Cichorium intybus*), Jerusalem artichoke (*Helianthus tuberosus*), artichoke (*Cynara scolymus*) and dandelions, and several cereals and grasses (Carpita *et al*., 1991; Darwen and John, 1989; Hellwege *et al*., 2000; Hendry, 1987; Van den Ende *et al*., 2000b; Wagner *et al*., 1983). Dandelion inulin crystals are clustered in parenchymal root cell vacuoles close to phloem tissues adjacent to laticifers (Javorsky, 1944; Van den Ende *et al*., 2000b) (Figure 1). The abundance of photoassimilates during the summer leads to the continuous accumulation of inulin during vegetative growth, as shown in chicory, Jerusalem artichoke and dandelions (van Arkel *et al*., 2012; Javorsky, 1944; Marx *et al*., 1997; Schorr‐Galindo and Guiraud, 1997; Van den Ende *et al*., 2000b). In autumn, declining levels of photoassimilates promote inulin degradation, and root tissue thus undergoes a sink‐to‐source transition (De Roover *et al*., 1999; Van den Ende and Van Laere, 1996; Van den Ende *et al*., 2000b).

The inulin metabolic pathway comprises three major enzymes representing glycosyl hydrolase (GH) family 32 (Henrissat, 1991; Verhaest *et al*., 2005) (Figure 1). The synthesis of inulin is catalysed by two fructosyltransferases (Edelman and Jefford, 1968; Lüscher *et al*., 1996). A sucrose: sucrose 1‐fructoslytransferase (1‐SST) (EC 2.4.1.99), which determines sink strength, produces the trisaccharide 1‐kestose by transferring a fructose residue from one sucrose molecule to another. Further fructose chain elongation is catalysed by fructan: fructan 1‐fructosyltransferase (1‐FFT) (EC 2.4.1.100). The degradation of inulin is catalysed by fructan 1‐exohydrolase (1‐FEH) (EC 3.2.1.80), which hydrolyses terminal fructose residues from fructan molecules sequentially until only a sucrose unit remains (De Roover *et al*., 1999; Edelman and Jefford, 1968). The degradation of sucrose is then catalysed by invertases (Sturm, 1999). In addition to its function as a rapidly accessible energy reservoir, inulin may protect the plant during drought or cold stress by stabilizing membranes (De Roover *et al*., 2000; Livingston and Henson, 1998; Pilon‐Smits *et al*., 1999; Valluru and Van den Ende, 2008).

In dandelion, the fact that inulin is localized adjacent to laticifers suggests that excess free sugars (e.g. fructose and sucrose) generated by inulin degradation could be used for the synthesis of IPP, increasing the production of triterpenes and NR. This is supported by the observation that wild‐grown *T. koksaghyz* can accumulate NR during dormancy, when the inulin level decreases (Ulmann, 1951). The opposite process was shown in *T. brevicorniculatum* plants that accumulate more inulin due to the inhibition of CPT activity by RNA interference, which resulted in lower levels of NR (Post *et al*., 2012). The excess IPP was first used for the formation of other isoprenoids such as sterols and pentacyclic triterpenes, and the eventual saturation of this relief pathway led to the accumulation of MVA pathway precursors, affecting upstream flux and redirecting carbon to the storage product inulin (Post *et al*., 2012).

It is important to understand the metabolic processes that affect NR synthesis, particularly in order to enhance the productivity of annually grown *T. koksaghyz* as a source of NR. Here, we provide evidence that a proportion of the energy supplied by inulin degradation is redirected to the biosynthesis of NR in wild‐type plants after sink‐to‐source transition in the roots and that the rubber content can therefore be improved by the overexpression of Tk 1‐FEH to promote further inulin degradation. Our study thus provides an appropriate basis for the breeding of rubber‐enriched dandelion varieties for industrial rubber production.

## Results and discussion

### Identification and characterization of *T. brevicorniculatum* and *T. koksaghyz* 1‐SST, 1‐FFT and 1‐FEH

We recently identified and characterized several dandelion genes involved in the synthesis of NR (Epping *et al*., 2015; Laibach *et al*., 2015; Post *et al*., 2012), but little is known about genes responsible for inulin metabolism. The full‐length *1‐SST*,* 1‐FFT* and *1‐FEH* cDNAs from *T. brevicorniculatum* and *T. koksaghyz* roots were therefore isolated based on known fructosyltransferase sequences and expressed sequence tag (EST) data.

*In silico* translation predicted open reading frames and molecular masses of 632 amino acids (aa) and 71.5 kDa for Tb1‐SST and Tk1‐SST, 622 aa and 69.6 kDa for Tb1‐FFT, and 622 aa and 69.7 kDa for Tk1‐FFT. The low predicted isoelectric points of pI 5.0 for Tb1‐SST and Tk1‐SST, and pI 5.2 for Tb1‐FFT and Tk1‐FFT, are common features among fructosyltransferases and 1‐FEHs (Lüscher *et al*., 2000; Sprenger *et al*., 1995; Van den Ende *et al*., 2001). Sequence alignment showed that Tb1‐SST and Tk1‐SST shared 100% pairwise identity, whereas Tb1‐FFT and Tk1‐FFT shared 97.8% pairwise identity. Each sequence shared more than 99% identity with its *T. officinale* orthologue and more than 79% identity with the corresponding proteins from chicory (Ci1‐SST AFB83198 and Ci1‐FFT AAD00558) and Jerusalem artichoke (Ht1‐SST CAA08812 and Ht1‐FFT CAA08811). Multiple sequence alignments using MUSCLE revealed the presence of three GH32 family‐specific conserved regions including the three catalytically active amino acids shown in bold: x‐x‐x‐**D**‐P‐D/N‐G; R**D**P; and **E**C (Altenbach and Ritsema, 2007; Altenbach *et al*., 2005; Edgar, 2004; Schroeven *et al*., 2008) (Figure S1). Furthermore, the fructosyltransferase‐specific motif x‐A/G‐Y/F was found in Tb1‐SST, Tk1‐SST, Tb1‐FFT and Tk1‐FFT (Altenbach *et al*., 2005; Lasseur *et al*., 2009).

The *in silico* translation of the amplified *Tb1‐FEH* and *Tk1‐FEH* cDNAs predicted proteins containing 581 aa, with molecular masses of 65.7 kDa and pI values of 5.8. SignalP predicted the presence of a 25‐residue N‐terminal signal peptide. Pairwise sequence alignment showed that Tb1‐FEH and Tk1‐FEH shared 98.6% identity and were also closely related to the chicory enzymes Ci1‐FEHIIa (CAC37922) with >90% identity and Ci1‐FEHIIb (CAC37923) with >88% identity. Both dandelion 1‐FEHs showed lower levels of identity with the chicory enzymes Ci1‐FEHI (CAC19366) with 52% identity, and an invertase (CAA72009) with 59% identity. The hydrolase‐specific W‐A/S/G‐W motif and the three conserved regions common to GH32 enzymes, including the three highly active amino acids mentioned above, were also found in Tb1‐FEH and Tk1‐FEH (Altenbach *et al*., 2005; Lasseur *et al*., 2009; Le Roy *et al*., 2007, 2008) (Figure S1).

The combined *in silico* data suggested that we had identified four fructosyltransferases as well as two 1‐FEHs that were suitable for further investigation.

### Analysis of inulin and NR metabolism in *T. koksaghyz* throughout the growing season

To gain an overview of inulin and NR metabolism in *T. koksaghyz* throughout the growing season, we analysed plants grown under near‐natural conditions outside the greenhouse with supplemental irrigation.

**Figure 2:**
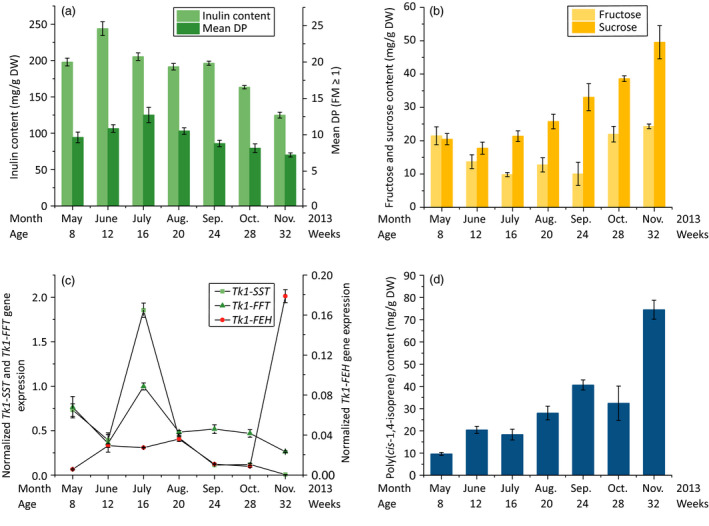
Analysis of inulin and NR metabolism in *T. koksaghyz* roots during the growing season. Every month, the roots of three randomly selected plants were harvested and pooled. Values are means ± SD of three independently sampled root extracts (a, b, d) or three cDNAs (c), each measured three times. (a) Inulin levels and mean DPs (HPLC), (b) fructose (HPLC) and sucrose (^1^H‐NMR) levels, (c) relative *Tk1‐SST*,* Tk1‐FFT* and *Tk1‐FEH* root mRNA levels (qPCR) normalized to the housekeeping gene *TkEf1*α and (d) poly(*cis*‐1,4‐isoprene) levels (^1^H‐NMR).

Approximately 200 mg/g DW inulin was present in the roots in May, but this increased to ~250 mg/g DW by June and the mean degree of polymerization (DP) increased from nine fructose molecules (FMs) in May to 12 by July (Figure 2a). During this time, the amounts of fructose (minimum 9.8 mg/g DW in July) and sucrose (minimum 17.7 mg/g DW in June), both of which are used to synthesize inulin and are released during its degradation, remained at low levels (Figure 2b). By November, nearly 50% of the stored inulin detected in July had degraded. Furthermore, the DP had decreased from 12 FMs in July to 7 FMs in November. This correlated with increasing fructose and sucrose levels. The amount of fructose increased strongly in October, reaching 24.3 mg/g DW by November, whereas sucrose levels increased to more than 35 mg/g DW in the autumn. Furthermore, throughout the growing season, the inulin level and DP precisely matched the *Tk1‐SST*,* Tk1‐FFT* and *Tk1‐FEH* expression profiles (Figure 2c). The increasing quantity and DP of inulin during the summer were accompanied by a parallel increase in *Tk1‐SST* and *Tk1‐FFT* gene expression. Both genes showed similar expression profiles throughout the growing season. From midsummer to autumn, the expression of the fructosyltransferases declined to nearly undetectable (*Tk1‐SST*) or low (*Tk1‐FFT*) levels in November, while the quantity and DP of inulin decreased. The correlation between fructosyltransferase expression and inulin levels has been already shown in chicory, Jerusalem artichoke and *T. officinale* (Koops and Jonker, 1996; Lüscher *et al*., 1996; Van den Ende and Van Laere, 1996; Van den Ende *et al*., 2000b). During the summer, an oversupply of photoassimilates seems to activate *Tk1‐SST* and *Tk1‐FFT*, promoting the accumulation of large amounts of high‐quality inulin in parenchymal cells adjacent to the root phloem (Javorsky, 1944; Van den Ende *et al*., 2000b). The down‐regulation of fructosyltransferases from midsummer to autumn resulted in the accumulation of sucrose in the root cells of *T. koksaghyz* (Figure 2b). Some of this sucrose is probably converted by invertases into fructose and glucose, leading to the high fructose level in October. Inulin degradation begins when the root undergoes a sink‐to‐source transition in late summer caused by photoperiodic changes from long‐day to short‐day conditions (De Roover *et al*., 1999; Van den Ende *et al*., 2000b). *Tk1‐FEH* was expressed at minimal levels during the summer months but was induced in the autumn and reached its maximum expression level in November. The down‐regulation of fructosyltransferases combined with strongly induced Tk1‐FEH activity contributed to the breakdown of inulin and increased the levels of fructose (to 24.3mg/g DW) and sucrose (to >50mg/g DW) by November (Figure 2a–c). Although the degradation of inulin addresses the need for a rapidly accessible energy supply, other factors may also play a significant role, such as overwintering and abiotic stress tolerance (Livingston and Henson, 1998; Tamura *et al*., 2014; Van den Ende and Van Laere, 1996; Van den Ende *et al*., 2000a).

Parenchymal cells containing inulin are located close to laticifers that produce NR, so it is possible that free sugars (e.g. fructose and sucrose) supplied by inulin degradation are used by the laticifers. The accumulation of poly(*cis*‐1,4‐isoprene) was first observed in young *T. koksaghyz* plants after the formation of laticifers. Eight‐week‐old plants contained only low levels of NR in the roots (Figure 2d). The poly(*cis*‐1,4‐isoprene) content in May was 9 mg/g DW, but this had risen to 40 mg/g DW by September. Although the lower photosynthetic rate in autumn provides lower amounts of substrate sugars for NR biosynthesis, the quantity of poly(*cis*‐1,4‐isoprene) nevertheless increased above 70 mg/g DW in November. The increasing level of poly(*cis*‐1,4‐isoprene) throughout the growing season was accompanied by the accumulation of fructose and sucrose in *T. koksaghyz* roots, indicating a connection between inulin degradation and the synthesis of NR. *H. brasiliensis* produces large amounts of high‐quality NR within its apoplastically separated laticifers, using sucrose as the exclusive precursor (Dusotoit‐Coucaud *et al*., 2009). Several sucrose transporters (HbSUT1A, HbSUT2A and HbSUT3) actively translocate sucrose from the apoplast into the laticifers (Dusotoit‐Coucaud *et al*., 2009, 2010; Tang *et al*., 2010). Additionally, the vacuolar release of sucrose via SUC4‐type transporters through the tonoplast was confirmed by the characterization of AtSUC4 in *Arabidopsis thaliana* roots (Endler *et al*., 2006; Sauer, 2007), whereas the vacuolar export of fructose is mediated by the fructose‐specific uniporter SWEET17 located on the tonoplast (Guo *et al*., 2014). In *T. koksaghyz*, similar transporters may enable the transport of fructose and sucrose generated by the degradation of inulin, moving them out of the vacuole, through the apoplast and into the laticifers.

### Functional characterization of ectopic Tk1‐FEH

**Figure 3:**
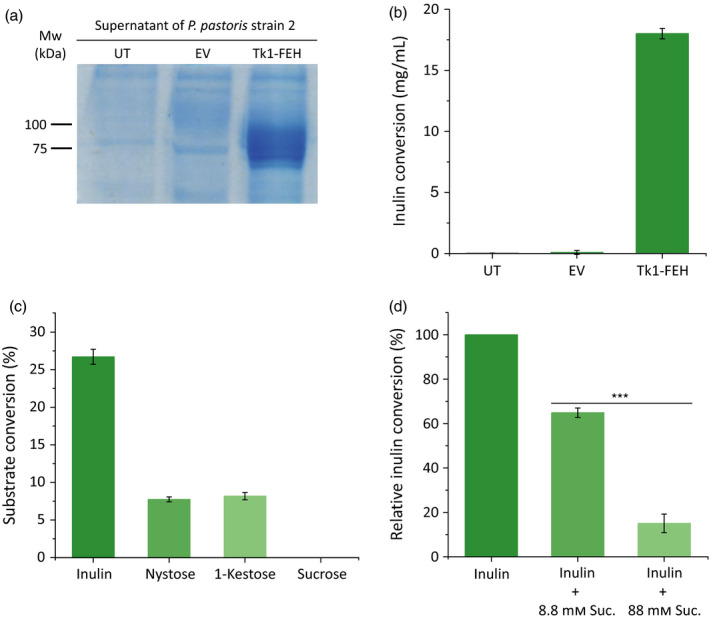
Analysis of ectopic Tk1‐FEH produced in *Pichia pastoris*. All investigations were carried out using dialysed supernatants from the expression cultures (a = 20 μL; b, c and d = 5 μL). Assays (b) and (d) were carried out using 5% chicory inulin dissolved in 150 μL McIlvaine buffer pH 5.1. (a) SDS‐PAGE analysis of proteins from untransformed *P. pastoris* (UT), *P. pastoris* carrying an empty pPinkα‐HC vector (EV) and *P. pastoris* transformed with Tk1‐FEH. (b) Inulin conversion of the *P. pastoris* cultures mentioned above. (c) Degradation of different substrates (14 mm each) by Tk1‐FEH measured for 3 h at 30 °C and pH 5.1. (d) Influence of sucrose on inulin degradation by Tk1‐FEH measured for 4 h at 30 °C (pH 5.1). Statistically significant differences are indicated by asterisks (*** *P *≤* *0.001; unpaired *t*‐test) (*n* = 3; mean ± SD).

Having identified a *T. koksaghyz* fructan 1‐exohydrolase and investigated its expression profile during the growing season, revealing a potential role in inulin degradation (Figure 2), we next studied its function by heterologous expression in the methylotrophic yeast *Pichia pastoris* strain 2. This host species does not express any fructosyltransferases, making it highly suitable for the production of recombinant 1‐FEHs (Xu *et al*., 2015). The native signal peptide was removed and replaced with an N‐terminal α‐mating factor signal peptide from *Saccharomyces cerevisiae* to ensure the secretion of Tk1‐FEH into the culture supernatant. The latter has a molecular mass of 20 kDa and is mostly removed before the recombinant protein is secreted from the yeast cell (Cereghino *et al*., 2002). SDS‐PAGE analysis revealed a ~75‐kDa band that was not present in the control samples (Figure 3a). Mass spectrometry revealed Tk1‐FEH‐specific peptide sequences covering 76.42% of the protein compared to the full‐length Tk1‐FEH used as target sequence (Table S1). Because glycosylation has previously been reported for heterologous 1‐FEH expression in yeast (Ueno *et al*., 2011; Xu *et al*., 2015), the glycosylation status of the recombinant Tk1‐FEH was checked by digestion with PNGase F. The enzyme removes *N*‐linked glycans and reduced the molecular mass of the recombinant Tk1‐FEH to the *in silico* predicted value of ~63 kDa without the native signal peptide (Figure S2).

The functionality of the recombinant Tk1‐FEH was determined by the conversion of different substrates followed by HPLC analysis. The conversion of inulin at levels greater than the negative control was only observed in samples containing the recombinant Tk1‐FEH (Figure 3b). The optimal pH of the recombinant Tk1‐FEH was 5.1 at 30 °C using chicory inulin as the substrate (Figure S2). The relatively acidic pH optimum is typical for vacuolar enzymes as already shown for 1‐FEHs from Jerusalem artichoke that are located in the vacuole closely associated with the tonoplast (Darwen and John, 1989). The substrate specificity of the recombinant Tk1‐FEH was determined by testing inulin, nystose, 1‐kestose and sucrose as substrates. Whereas 27% of the inulin (DP ≈ 21) and ~8% of the nystose and 1‐kestose were converted by Tk1‐FEH, sucrose was unaffected (Figure 3c). Furthermore, less inulin was converted when inulin and sucrose were used as combined substrates, suggesting that sucrose acted as a dose‐dependent inhibitor as shown for other plant 1‐FEHs (Lothier *et al*., 2007; Xu *et al*., 2015) (Figure 3d). Compared to the activity displayed with inulin alone as the substrate, the presence of 8.8 mm sucrose reduced the conversion efficiency to 65%, and the presence of 88 mm sucrose reduced the conversion efficiency to 15%. These data, together with the lack of activity against sucrose as the sole substrate, confirmed that Tk1‐FEH is not an invertase. We thus propose that Tk1‐FEH is a functional 1‐FEH with a substrate preference for inulin with a DP > 4.

### Overexpression of *Tk1‐FEH* results in the degradation of inulin

The functional analysis of Tk1‐FEH was also carried out *in planta* by cloning the full‐length *Tk1‐FEH* cDNA under the control of the constitutive CaMV35S promoter and expressing it in *T. brevicorniculatum* and *T. koksaghyz*. *T. brevicorniculatum* is apomictic and therefore produces genetically homogenous progeny, which facilitates the characterization of transgenic plants. Transformation resulted in more than 10 independent transgenic lines in both species.

**Figure 4:**
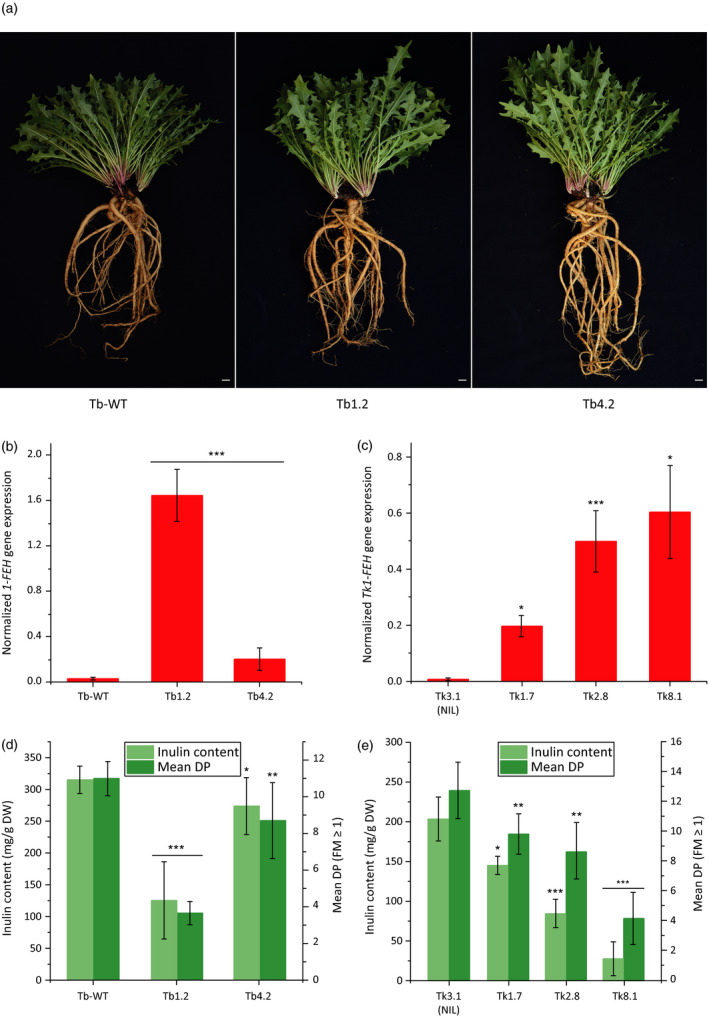
Overexpression of *Tk1‐FEH* in dandelion leads to inulin degradation. Investigations were carried out using root material from 18‐week‐old *T. brevicorniculatum* (*n* = 6–9) and *T. koksaghyz* (*n* = 3–6) plants (T1 generation). Asterisks indicate statistically significant differences between transgenic plant lines and their controls (* *P *≤* *0.05; ** *P *≤* *0.01; and *** *P *≤* *0.001; unpaired *t*‐test or Mann–Whitney *U* test). (a) Eight‐month‐old *T. brevicorniculatum* plants overexpressing *Tk1‐FEH* (Tb1.2 centre; Tb4.2 right) and their control (Tb‐WT left) (scale bar = 2 cm). (b and c) *1‐FEH* mRNA levels in roots of *T. brevicorniculatum* (b) and *T. koksaghyz* (c) plants determined by qPCR and normalized using the housekeeping genes *TbEf1*α and *TkEf1*α. (d and e) Inulin content and mean DP in *T. brevicorniculatum* (d) and *T. koksaghyz* (e) plants determined by HPLC.

Transgene expression in the T0 generation was confirmed by PCR, and quantitative analysis by real‐time PCR (qPCR) revealed two transgenic *T. brevicorniculatum* plants (Tb1.2 and Tb4.2) and four *T. koksaghyz* plants (Tk1.7, Tk2.8, Tk3.1 and Tk8.1) with high, moderate or low levels of transgene expression (data not shown). These were used to generate T1 lines for subsequent analysis. Plants Tb1.2 and Tb4.2 were used to produce seven (Tb4.2) and nine (Tb1.2) T1 offspring, which were compared to nine wild‐type *T. brevicorniculatum* control plants. The four transgenic *T. koksaghyz* plants (Tk1.7, Tk2.8, Tk3.1 and Tk8.1) were pollinated with one *T. koksaghyz* wild‐type plant for seed generation, resulting in three (Tk1.7 and Tk8.1), four (Tk3.1) and six (Tk2.8) T1 plants for further investigation. The plants of both species were grown under greenhouse conditions and harvested after 18 weeks for the detailed analysis of development, gene expression and metabolite composition. Phenotypic analysis of the transgenic lines revealed that transgene expression had no impact on development in terms of biomass, flowering, seed setting or germination. The phenotypes of T1 plants from *T. brevicorniculatum* lines Tb1.2 and Tb4.2 are shown as representative examples in Figure 4a.

*1‐FEH* gene expression was analysed in more detail by qPCR, revealing a significantly higher mean relative expression level in Tb1.2 (57‐fold higher than the wild‐type control) and Tb4.2 (sevenfold higher than the wild‐type control) (Figure 4b). Transgenic *T. koksaghyz* lines Tk1.7, Tk2.8 and Tk8.1 expressed *1‐FEH* at high levels, and the transgenic near isogenic line (NIL) Tk3.1 was used as a control because the *1‐FEH* expression level was similar to wild type (Figure 4c). *1‐FEH* gene expression was 21.5‐fold higher in line Tk1.7, 56.5‐fold higher in line Tk2.8 and 68.3‐fold higher in line Tk8.1, in each case compared to the control (Tk3.1). The quantitative analysis of *1‐SST* and *1‐FFT* gene expression revealed no significant differences between the transgenic lines and their controls, indicating that *1‐FEH* does not influence the transcription of fructosyltransferases in dandelion (Figure S3).

Although there were no phenotypic differences between transgenic lines and their controls, metabolic analysis revealed a significant reduction in the amount of inulin in the transgenic lines: 125 mg/g DW in line Tb1.2 and 274 mg/g DW in line Tb4.2 compared to 315 mg/g DW in wild‐type plants (Figure 4d). Additionally, the average inulin DP in both *T. brevicorniculatum* lines differed significantly from the wild‐type value of 11 FMs, that is four FMs in line Tb1.2 and nine FMs in line Tb4.2. *T. koksaghyz* lines Tk1.7, Tk2.8 and Tk8.1 also produced significantly lower amounts of inulin with lower average DPs than line Tk3.1 (Figure 4e). The amount and DP of inulin in all the *T. brevicorniculatum* and *T. koksaghyz* lines negatively correlated closely with the relative transgene expression level.

### Inulin degradation influences triterpene and NR levels in dandelion

**Figure 5:**
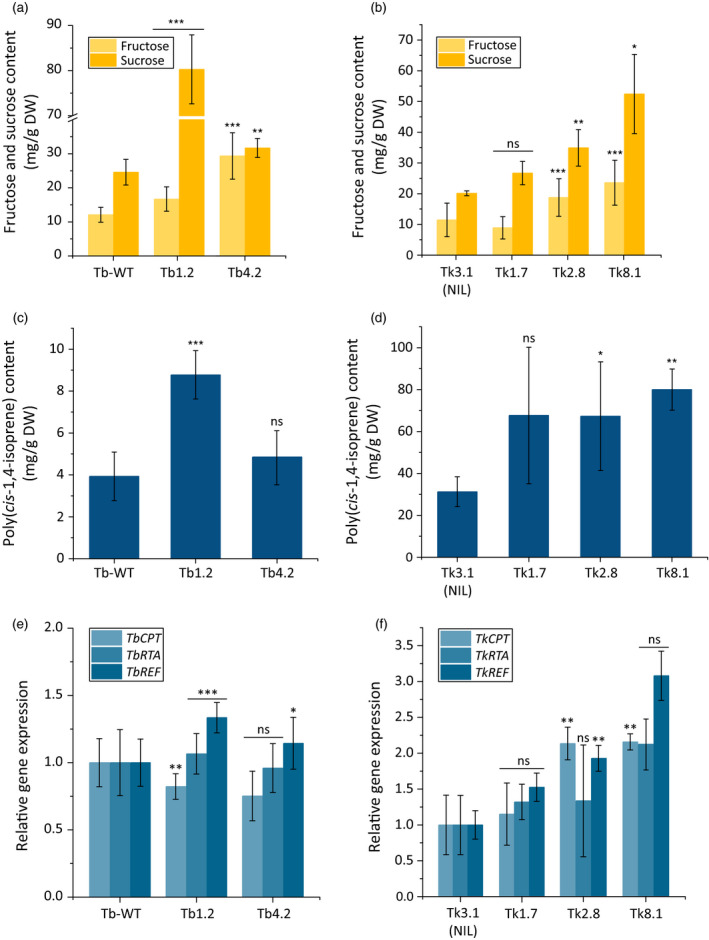
Overexpression of *Tk1‐FEH* in dandelion promotes sugar accumulation and NR biosynthesis. Asterisks indicate statistically significant differences between transgenic plant lines and their controls (* *P *≤* *0.05; ** *P *≤* *0.01; and *** *P *≤* *0.001; unpaired *t*‐test or Mann–Whitney *U* test). *T. brevicorniculatum*:* n* = 6–9 and *T. koksaghyz*:* n* = 3–6 plants (T1). (a and b) Fructose (HPLC) and sucrose (^1^H‐NMR) levels in *T. brevicorniculatum* (a) and *T. koksaghyz* (b) plants. (c and d) Poly(*cis*‐1,4‐isoprene) levels (^1^H‐NMR) in *T. brevicorniculatum* (c) and *T. koksaghyz* plants (d). (e and f) Endogenous *CPT*, *RTA* and *REF* mRNA levels (qPCR) in *T. brevicorniculatum* (e) and *T. koksaghyz* (f) roots, normalized using the housekeeping genes *TbEf1*α and *TkEf1*α.

We next analysed in both dandelion species the increase in fructose and sucrose levels as a result of inulin degradation after 18 weeks. Both sugars increased in abundance in the transgenic *T. brevicorniculatum* and *T. koksaghyz* lines compared to control lines, mirroring the *1‐FEH* expression levels (Figure 5a and b). The amount of sucrose in lines Tb1.2 and Tb4.2 was 48 and 24 mg/g DW, respectively, compared to the wild‐type level of 16 mg/g DW. In the transgenic *T. koksaghyz* lines, the sucrose level reached a maximum of 27 mg/g DW (line Tk8.1) compared to 15 mg/g DW in the control line Tk3.1. The fructose levels in both species were affected in a similar manner (Figure 5a and b). The free sugar levels could potentially promote among other pathways the biosynthesis of different isoprenoid end products in the roots, such as triterpenes and NR. Therefore, we determined the quantity of sterols, pentacyclic triterpenes and poly(*cis*‐1,4‐isoprene) in *T. brevicorniculatum* and *T*. *koksaghyz* (Table 1, Figure 5c and d). GC‐MS analysis revealed significantly higher levels of sterols (campesterol, stigmasterol and sitosterol) and a remarkable increase in pentacyclic triterpenes such as taraxasterol, taraxerol, α‐amyrin, β‐amyrin and lupeol, in all the transgenic lines compared to the corresponding controls.

**Table 1:** Triterpene content of *Taraxacum* plants overexpressing *Tk1‐FEH*

Plant lines	Total triterpenes ± SD (mg/g DW)	Sterols ± SD (mg/g DW)	Pentacyclic triterpenes ± SD (mg/g DW)
Tb‐WT	12.57 (±2.27)	2.10 (±0.33)	10.47 (±1.97)
Tb1.2	24.32 (±5.20)***	3.54 (±0.75)***	20.78 (±4.49)***
Tb4.2	17.55 (±1.92)***	2.73 (±0.26)***	14.81 (±1.77)***
Tk3.1 (NIL)	13.72 (±1.70)	2.80 (±0.39)	10.91 (±1.79)
Tk1.7	19.15 (±0.95)**	3.70 (±0.10)*	15.45 (±1.04)*
Tk2.8	18.10 (±5.54)^ns^	3.72 (±0.47)*	14.38 (±5.20)^ns^
Tk8.1	19.44 (±2.21)*	4.51 (±0.21)**	14.93 (±2.23)*

Asterisks indicate statistically significant differences between the transgenic lines and their corresponding controls (*P ≤* *0.05; **P ≤* *0.01; and ***P ≤* *0.001; ns = nonsignificant; unpaired *t*‐test).

The quantity of poly(*cis*‐1,4‐isoprene) correlated in both species with the *1‐FEH* expression level. In *T. brevicorniculatum* line Tb1.2, the amount of poly(*cis*‐1,4‐isoprene) was almost 10 mg/g DW, compared to ≤4 mg/g DW in wild‐type plants (Figure 5c). In contrast, although the average quantity of poly(*cis*‐1,4‐isoprene) also increased in line Tb4.2, the increase compared to wild‐type plants was not statistically significant (4.8 mg/g DW). These data concur with the intermediate level of *1‐FEH* gene expression and the lower impact on inulin and sugar (fructose and sucrose) levels in those plants (Figures 4b, d and 5a). *T. koksaghyz* produces up to 10 times more NR in the roots than *T. brevicorniculatum*, as shown by the poly(*cis*‐1,4‐isoprene) content of 32.6 mg/g DW in control line Tk3.1. Notably, the overexpression of *1‐FEH* increased the quantity of poly(*cis*‐1,4‐isoprene) even in the transgenic *T. koksaghyz* lines, reaching a maximum mean value of 80 mg/g DW in line Tk8.1 (Figure 5d). Furthermore, we found that the amount of poly(*cis*‐1,4‐isoprene) correlated with the expression levels of two genes encoding enzymes in the rubber transferase complex (*CPT* and *RTA*) and the rubber elongation factor gene (*REF*) encoding a rubber particle‐stabilizing protein (Epping *et al*., 2015; Laibach *et al*., 2015). A significant increase in the expression of *CPT*,* RTA* and *REF* individually or together was observed in lines Tb1.2, Tk2.8 and Tk8.1 compared to the control plants (Figure 5e and f).

**Figure 6:**
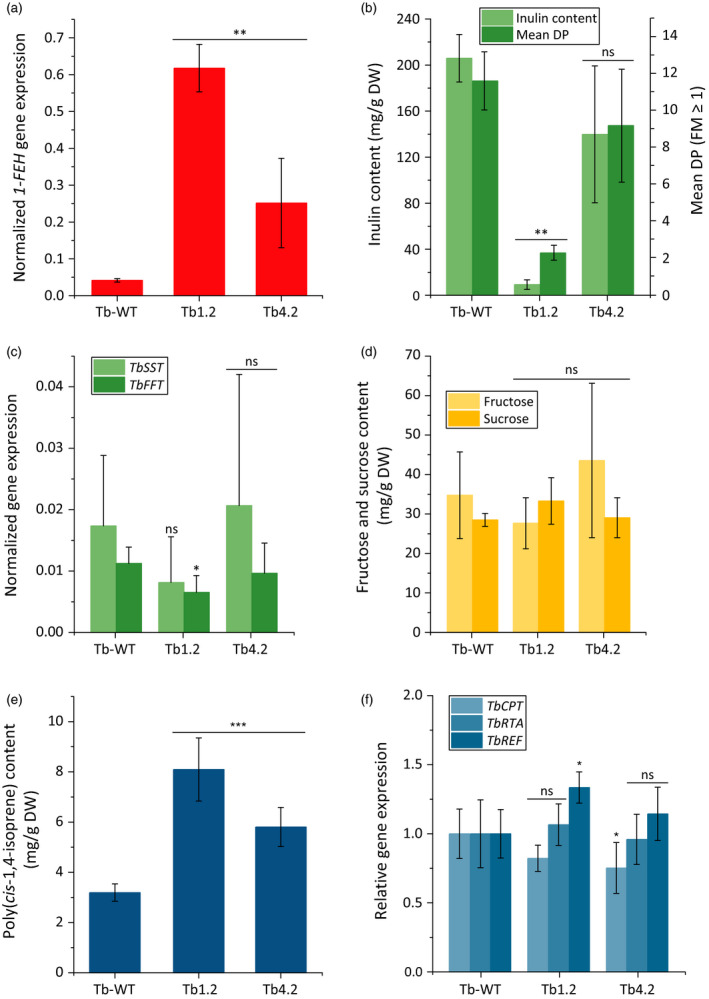
Effect of *Tk1‐FEH* overexpression in 40‐week‐old *T. brevicorniculatum* plants. Investigations were performed with root material from 40‐week‐old *T. brevicorniculatum* (*n* = 5) plants (T1 generation) grown in the greenhouse. Asterisks indicate statistically significant differences between transgenic plant lines and their wild‐type control (*P ≤* *0.05; ** *P *≤* *0.01; and ns = nonsignificant; Mann–Whitney *U* test). The *1‐FEH* (a), *Tb‐1‐SST* and *Tb1‐FFT* (c) and *TbCPT*,* TbRTA* and *TbREF* (f) mRNA levels in roots of *T. brevicorniculatum* plants determined by qPCR and normalized using the housekeeping gene *TbEf1*α. (b) Inulin content and mean DP in *T. brevicorniculatum* plants determined by HPLC. (d) Fructose (HPLC) and sucrose (^1^H‐NMR) levels in *T. brevicorniculatum* plants. (e) Poly(*cis*‐1,4‐isoprene) levels (^1^H‐NMR) in *T. brevicorniculatum* plants (f) Relative *TbCPT, TbREF* and *TbRTA* mRNA levels in roots of *T. brevicorniculatum* plants determined by qPCR and normalized using *TbEf1α*.

In addition to 18‐week‐old plants, we analysed 40‐week‐old *T. brevicorniculatum* plants overexpressing *1‐FEH* to investigate the metabolic impact of inulin degradation at the late harvesting stage in a normal cultivation period. As above, none of the transgenic plants showed any phenotypic aberrations (data not shown), but we observed *1‐FEH* dosage‐dependent differences in the inulin, poly(*cis*‐1,4‐isoprene) and triterpene levels compared to wild‐type plants (Figure 6 and Table S2). In contrast to 18‐week‐old plants, a further increase in the rubber content was evident solely in line Tb4.2 and not in line Tb1.2 (Figures 5c and 6e). This may reflect the fact that the inulin content in 18‐week‐old Tb1.2 plants was already low (~125 mg/g DW; Figure 4d) due to the strong expression of *1‐FEH* (Figure 4b). In contrast, the inulin content in line Tb4.2 (~275 mg/g DW; Figure 4d) was only minimally affected compared to wild‐type plants due to the low level of *1‐FEH* expression (Figure 4b). Therefore, the inulin pool in line Tb4.2 can still be converted into rubber as indicated by the increase in the rubber content of 40‐week‐old plants (Figure 6e). Additionally, a significant portion of the inulin appeared to be metabolized for other housekeeping functions, reducing the inulin content of the 40‐week‐old wild‐type plants by ~100 mg/g DW (Figure 6b) compared to 18‐week‐old plants (Figure 4d). This was also be evident in the transgenic lines. The *de novo* biosynthesis of inulin in older plants appears unlikely due to the low level of the enzymes 1‐SST and 1‐FFT (Figure 6c). The wild‐type and transgenic lines contained similar amounts of sucrose and fructose (although the fructose content of line Tb4.2 was slightly higher) mainly representing the accumulation of photoassimilates (Figure 6d). The expression of the NR biosynthesis genes *CPT*,* RTA* and *REF* was comparable in all lines (Figure 6f).

Taken together, the overexpression of *Tk1‐FEH* showed that the free sugars derived from the degradation of inulin promote isoprenoid biosynthesis among other pathways in the roots without any trade‐offs in terms of plant fitness. The higher levels of poly(*cis*‐1,4‐isoprene) already detected in 18‐week‐old plants but also present in 40‐week‐old plants (Tb4.2) may offer the opportunity to select plants with defined breeding characteristics in terms of the optimal harvesting time. Varieties with a high basal expression level of *1‐FEH* could be used for early harvesting (in late summer) potentially allowing more crop rotation. In contrast, plants with moderate increases in *1‐FEH* expression would reach their maximum NR content later in the year and would allow the harvesting of more biomass, thus achieving an overall higher yield per acre in autumn. Furthermore, we found that the inulin and NR contents in *T. koksaghyz* are influenced not only by changes in *Tk1‐FEH* gene expression during the growing season, but also by the basal expression level, leading to natural variation in rubber productivity in different dandelion accessions. Our data indicate that the inulin content and *1‐FEH* expression level are important biochemical and genetic markers that can be used to select rubber‐enriched annual *T. koksaghyz* varieties that can be used as a commercially feasible crop for future industrial NR production.

## Experimental procedures

### Plant material and cultivation

*Taraxacum koksaghyz* wild‐type plants, used for the analysis of inulin and NR metabolism throughout the growing season, were sown early in March 2013 and cultivated as previously described (Laibach *et al*., 2015). Early in May, the young plants were placed outside the greenhouse with supplemental irrigation. On day 15 of every month, three plants were picked randomly, and the roots were harvested, quick‐frozen, lyophilized and ground to powder. Plants used in the *Tk1‐FEH* overexpression experiments were sown in the greenhouse under the conditions described by Laibach *et al*. (2015). The plants were harvested 18 or 40 weeks after sowing, and the roots were processed as described above.

### Amplification of full‐length *1‐SST*,* 1‐FFT* and *1‐FEH* cDNA sequences

The *1‐SST* and *1‐FFT* coding sequences were amplified from *T. brevicorniculatum* and *T. koksaghyz* root cDNA using the oligonucleotide combinations *1‐SST‐SalI‐fwd*/*1‐SST‐NheI‐rev* and *1‐FFT‐NotI‐fwd*/*1‐FFT‐XbaI‐rev* (Table S3), respectively. All oligonucleotides were based on cDNA sequences from *T. officinale 1‐SST* (AJ250634) and *1‐FFT* (AJ829549), as well as *T. officinale 1‐FFT*‐specific EST data (DY802367). The *Tb1‐FEH* and *Tk1‐FEH* coding sequences were amplified from root cDNA by 3′‐RACE PCR using *1‐FEH‐GSP1‐3′* based on *T. officinale* EST data (DY815781) as the gene‐specific oligonucleotide (Table S3). The 3′‐RACE PCR was carried out as previously described (Schmidt *et al*., 2010a). The partial *1‐FEH* sequences were completed by genome walking using the Universal GenomeWalker^™^ Kit (Clontech, Saint‐Germain‐en‐Laye, France) according to the manufacturer's instructions, with *1‐FEH‐GW1* and *1‐FEH‐GW2* as gene‐specific oligonucleotides (Table S3).

### Total RNA extraction and cDNA synthesis

Total RNA was extracted from the roots of wild‐type *T. koksaghyz* plants using the NucleoSpin^®^ RNA Plant kit (Macherey‐Nagel, Düren, Germany) according to the manufacturer's instructions. Total root RNA from other *Taraxacum* plants was extracted using the innuPREP RNA Mini Kit (Analytik Jena, Jena, Germany) according to the manufacturer's instructions. The cDNA was synthesized using the all‐in‐one PrimeScript^™^ Reverse Transcriptase Master Mix from TAKARA (Clontech) according to the manufacturer's instructions.

### Gene expression analysis by quantitative real‐time PCR

Quantitative real‐time PCR (qPCR) was carried out as previously described (Laibach *et al*., 2015). *T. koksaghyz* wild‐type samples (harvested from May to November 2013) represented nine technical replicates of three individual cDNAs synthesized from one total root RNA sample of three pooled plants. Samples for all other qPCRs represented three technical replicates of root material from one individual plant, later pooled as 3–9 biological replicates. The housekeeping gene elongation factor 1α (*Ef1*α) was used for the normalization of gene expression in *T. brevicorniculatum* (*TbEF1*α) and *T. koksaghyz* (*TkEF1*α). Oligonucleotides used to measure gene expression levels are listed in Table S3. Conserved sequences were used for each oligonucleotide so that they were suitable for both *T. brevicorniculatum* and *T. koksaghyz*. Quantitative PCR data were analysed as previously described (Laibach *et al*., 2015). Species‐ and root‐specific oligonucleotide efficiencies were calculated as previously reported (Table S4).

### Cloning the *Tk1‐FEH* overexpression constructs

The full‐length *Tk1‐FEH* cDNA was amplified using oligonucleotides *1‐FEH‐XhoI‐fwd* and *1‐FEH‐XbaI‐rev* (Table S3) and inserted into the expression vector pLab12.10 using the restriction sites XhoI and XbaI (Xing *et al*., 2014). The final construct (pLab12.10‐CaMV35SP‐Tk1‐FEH‐CaMV35ST) was validated by sequencing.

### *Agrobacterium*‐mediated transformation of *Taraxacum* spp.

The transformation of *T. brevicorniculatum* and *T. koksaghyz* plants was carried out as previously described with slight modifications (Post *et al*., 2012). *T. koksaghyz* leaf discs were incubated on callus induction medium containing 400 mg/L amoxicillin, and shoot induction medium was supplemented with 1 mg/L kinetin, 100 μg/L indole acetic acid and 200 mg/L amoxicillin. Root induction in *T. koksaghyz* was triggered by placing the shoots on shoot induction medium supplemented with 400 mg/L amoxicillin.

### Analysis of inulin levels and DP in dandelion roots by HPLC

Ground root material was boiled for 18 h at 85 °C using HPLC‐grade water (1 : 10 w/v) as the solvent. For clarification, the inulin‐containing extract was centrifuged at 5000 **g**. After 20 min, 500 μL of the supernatant was incubated with 500 μL 20 mm acetate buffer (pH 4.15) for 2 h at 55 °C, shaking at 700 r.p.m. Another 500 μL of the supernatant was incubated with 490 μL 20 mm acetate buffer (pH 4.15) and 10 μL (110 U/L) *Aspergillus niger* inulinase (Sigma‐Aldrich, St. Louis, MI) dissolved in the same buffer. Both reactions were stopped by adding 1 mm EDTA (pH 8.0), followed by centrifugation at 13 000 **g** for 2 min. To verify the activity of inulinase, we also digested 20 mg/mL chicory inulin dissolved in HPLC‐grade water. Fructose, glucose and sucrose levels in each sample were determined at 40 °C by HPLC using the RID‐10A refractive index detector (Shimadzu, Duisburg, Germany) and the Asahipak NH2P‐50 4E column (Shodex^™^, Mainz, Germany). Acetonitrile with HPLC‐grade water (75 : 25 v/v) was used as the mobile phase with a flow rate of 1 mL/min. The peak areas of 0.2–75 mg/mL fructose, glucose and sucrose standards were used for calibration and to establish detection limits. The inulin content was determined by comparing the fructose, glucose and sucrose levels of undigested and digested samples of the same inulin extract as previously described with slight modifications (Hahn *et al*., 2016).

The mean DP of inulin was determined by considering sucrose as the smallest possible sugar molecule resulting from the degradation of inulin either by 1‐FEH or by inulinase. Therefore, sucrose was not considered in the determination of the mean DP, which was calculated as previously reported (Hahn *et al*., 2016).

### Determination of poly(*cis*‐1,4‐isoprene) levels by ¹H‐NMR spectroscopy

Poly(*cis*‐1,4‐isoprene) levels were analysed by ¹H‐NMR using 150–200 mg of ground root material supplemented with 1500 μL of a mixture containing 10% toluene‐d8, tetramethylsilane and 16 mm 2,6‐dimethoxyphenol (DMOP) as internal standards. Extraction was carried out for 16 h at 20 °C, shaking at 1000 r.p.m. After centrifugation (21 000 **g**, 110 min), 600 μL of the supernatant was analysed using a Bruker Avance III 400 MHz spectrometer with a 5‐mm broadband inverse (BBI) probe head (Bruker, Billerica, MA) at 298 K. All data were acquired using a one‐dimensional ¹H‐NMR pulse program with 90° pulse and a relaxation delay of 20 s. The raw data were processed including the correction of the phase and baseline. For quantitative analysis, the C5 methyl signal for poly(*cis*‐1,4‐isoprene) was integrated at 1.75 ppm and the methyl signal of DMOP at 3.34 ppm. The quality control for each run was performed by checking the integrals of DMOP against calibrator samples. A control sample with a known poly(*cis*‐1,4‐isoprene) content was also analysed in each run.

### Determination of sucrose levels by ¹H‐NMR spectroscopy

Sucrose was extracted from 100 mg of ground root material by adding 1500 μL 0.1 m phosphate buffer (pH 6.8) containing 5% D_2_O and 1 mm trimethylsilylpropanoic acid (TSP) as an internal standard. Extraction was carried out for 1 h at 85 °C and 16 h at 20 °C, shaking at 1000 r.p.m, and 0.5 h at 85 °C again, followed by centrifugation (21 000 **g**, 10 min). Analysis of the supernatant (600 μL) was performed as described above at 310 K, with a 30° pulse and a relaxation delay of 15 s. Data analysis was carried out as above. The signal of the internal TSP standard integrated at 0.0 ppm and the signal at 5.44 ppm for sucrose (anomeric glucose proton) were used to quantify the sucrose content. Quality control was performed by comparing the integral of TSP against calibrator samples.

### Extraction of triterpenes and GC‐MS analysis

Triterpenes were extracted as described by Post *et al*. (2012) using 250 μg betulin as the internal standard. Hexane phases were pooled and evaporated to dryness, and the residue was dissolved in 1 mL acetone overnight before analysis by GC‐MS as described by Xing *et al*. (2014).

### Heterologous expression in *Pichia pastoris*

The *Tk1‐FEH* sequence was amplified from pLab12.10‐CaMV35SP‐Tk1‐FEH‐CaMV35ST using the oligonucleotide combination *Tk1‐FEH‐fwd* and *Tk1‐FEH‐KpnI‐rev* (Table S3). The product was introduced into the pPinkα‐HC vector at the StuI and KpnI sites, followed by transformation according to the manufacturer's introductions (PichiaPink^™^ Expression System; Thermo Fisher Scientific, Darmstadt, Germany). Transgene integration was checked by colony PCR using the oligonucleotide combination *5'AOX* and *1‐FEH‐RT‐rev*. Large‐scale expression of the recombinant Tk1‐FEH was achieved using PichiaPink^™^ strain 2, with untransformed and empty vector controls. Supernatants were separately frozen in liquid nitrogen and stored at −80 °C.

Purification and concentration of the recombinant Tk1‐FEH from the supernatants were achieved using a combination of precipitation with 80% (v/v) ammonium sulphate and dialysis. The precipitated proteins were dissolved in 2 mL McIlvaine buffer (pH 6.0). The solution was dialysed against 1 L McIlvaine buffer (pH 6.0) at 4 °C for 18 h using a membrane with a 14 kDa cut‐off. The dialysed samples were quick‐frozen and stored at −20 °C before protein separation and visualization by SDS‐PAGE (Laemmli, 1970), deglycosylation by Remove‐iT^®^ PNGase F (New England Biolabs, Ipswich, MA) or enzymatic assays.

### HPLC‐coupled end point determination of Tk1‐FEH characteristics

The end point enzymatic assays described below were followed by HPLC analysis to measure residual glucose, fructose and sucrose concentrations. The final substrate concentrations (mg/mL) were determined by the addition of the measured sugars minus one tenth of fructose. Based on these standards, the concentrations of glucose, fructose and sucrose were found to be 0.3–100 mg/mL. All reactions were stopped by enzyme denaturation at 80 °C for 5 min. After centrifugation (13 000 **g**, room temperature, 2 min), the samples were measured for HPLC as described above.

All assays were carried out using 5 μL of the purified supernatants containing 8.46 μg of total protein, determined using the Bradford method (Bradford, 1976). Control assays were prepared using supernatant from untransformed yeast, empty vector controls or yeast transformed with Tk1‐FEH mixed with 150 μL 5% (w/v) chicory inulin (14 mm; DP ≈ 21). All samples were incubated for 3 h at 30 °C, shaking at 700 r.p.m. Substrate conversion was analysed by mixing the supernatants of yeasts transformed with the empty vector or Tk1‐FEH with 200 μL 14 mm chicory inulin (DP ≈ 21), nystose, 1‐kestose or sucrose dissolved in McIlvaine buffer (pH 5.1). The reactions were stopped after 3 h at 30 °C, shaking at 700 r.p.m. Substrate concentrations were determined after subtracting the empty vector control values and were presented as the percentage conversion of the applied substrates. The influence of sucrose on inulin degradation was determined by mixing 5 μL of the supernatant including Tk1‐FEH or 5 μL McIlvaine buffer (pH 5.1) with 150 μL 5% (w/v) chicory inulin in McIlvaine buffer (pH 5.1) supplemented with either 15 μL 1 m sucrose in McIlvaine buffer (pH 5.1), 15 μL 100 mm sucrose in McIlvaine buffer (pH 5.1) or 15 μL McIlvaine buffer (pH 5.1). All samples were incubated for up to 4 h at 30 °C, shaking at 700 r.p.m. Inulin conversion was presented after subtracting the values of the buffer controls with no enzyme.

### Characterization of recombinant Tk1‐FEH by mass spectrometry; pH‐dependent HPLC‐coupled end point determination of 1‐FEH characteristics; Statistical analysis

See Supplementary methods.

## Conflict of interest

The authors declare no conflicts of interest.

## Supporting information


**Figure S1.** Partial multiple sequence alignment of several GH32‐family proteins.
**Figure S2.** Characterization of recombinant Tk1‐FEH.
**Figure S3.** *1‐SST* and *1‐FFT* gene expression levels in 18‐week‐old *T. brevicorniculatum* and *T. koksaghyz* plants overexpressing *Tk1‐FEH*.
**Table S1**. Analysis of Tk1‐FEH expression in yeast cultures by mass spectrometry.
**Table S2.** Triterpene content of 40‐week‐old *T. brevicorniculatum* plants overexpressing *Tk1‐FEH*.
**Table S3.** List of oligonucleotides used in this study.
**Table S4.** Oligonucleotide efficiencies for qPCR.
**Data S1.** Supplementary methods.
