# Supplementary material for: Development of rubber‐enriched dandelion varieties by metabolic engineering of the inulin pathway
Source: Plant Biotechnol J. 2017 Feb 9;15(6):740–53. doi: 10.1111/pbi.12672 (PMC5425391; doi:10.1111/pbi.12672)
Supplement: Supplementary file 1 — Figure S1. Partial multiple sequence alignment of several GH32‐family proteins. Figure S2. Characterization of recombinant Tk1‐FEH. Figure S3. 1‐SST and 1‐FFT gene expression levels in 18‐week‐old T. brevicorniculatum and T. koksaghyz plants overexpressing Tk1‐FEH. Table S1. Analysis of Tk1‐FEH expression in yeast cultures by mass spectrometry. Table S2. Triterpene content of 40‐week‐old T. brevicorniculatum plants overexpressing Tk1‐FEH. Table S3. List of oligonucleotides used in this study. Table S4. Oligonucleotide efficiencies for qPCR. Data S1. Supplementary methods. [file PBI-15-740-s001.docx]

(a)

1-SST[*Helianthus tuberosus*]CAA08812 YHFQPDKNFIS**D**PDGPMYHMGWYHLFYQYNPQSAIWGNITWGHSVSKDMINWFHLPFAMV 156

1-SST[*Cichorium intybus*]AFB83198.1 YHFQPDKNFIS**D**PDGPMYHMGWYHLFYQYNPESAIWGNITWGHSVSRDMINWFHLPFAMV 169

1-SST[*Taraxacum koksaghyz*] YHFQPDKNFIS**D**PDGPMYHMGWYHLFYQYNPESAIWGNITWGHSISRDMINWFHLPFAMV 160

1-SST[*Taraxacum officinale*]CAB60153 YHFQPDKNFIS**D**PDGPMYHMGWYHLFYQYNPESAIWGNITWGHSISRDMINWFHLPFAMV 160

1-FFT[*Helianthus tuberosus*]CAA08811 FHFQPAKNFIY**D**PDGQLFHMGWYHMFYQYNPYAPVWGNMSWGHSVSKDMINWYELPVAMV 150

1-FFT[*Taraxacum koksaghyz*] YHFQPAKNFIY**D**PNGPLFHMGWYHLFYQYNPYAPIWGNMSWGHAVSKDMIHWFELPVAIV 143

1-FFT[*Cichorium intybus*]AFB83199.1 YHFQPAKNFIY**D**PNGPLFHMGWYHLFYQYNPYAPIWGNMSWGHAVSKDMVNWFELPVALT 143

:**** **** ******:* ::******:****** :.:***::***::*.**::*: **.*:.

1-SST[*Helianthus tuberosus*]CAA08812 PDHWYDIEGVMTGSATVLPNGQIIMLYSGNAYDLSQVQCLAYAVNSSDPLLIEWKKYEGN 216

1-SST[*Cichorium intybus*]AFB83198.1 PDHWYDIEGVMTGSATVLPNGQIIMLYTGNAYDLSQLQCLAYAVNSSDPLLLEWKKYEGN 228

1-SST[*Taraxacum koksaghyz*] PDHWYDIEGVMTGSATMLPDGQIIMLYTGNAYDLAQLQCLAYAVNSSDPLLLEWKKYEGN 220

1-SST[*Taraxacum officinale*]CAB60153 PDHWYDIEGVMTGSATMLPDGQIIMLYTGNAYDLAQLQCLAYAVNSSDPLLLEWKKYEGN 220

1-FFT[*Helianthus tuberosus*]CAA08811 PTEWYDIEGVLSGSTTVLPNGQIFALYTGNANDFSQLQCKAVPVNLSDPLLIEWVKYEDN 210

1-FFT[*Taraxacum koksaghyz*] PTEWYDIEGVLSGSTTALPNGQIFALYTGNAKDFSQLQCKAVPLNASDPLLVEWVKYEDN 203

1-FFT[*Cichorium intybus*]AFB83199.1 PTEWYDFEGVLSGSTTVLPNGQIFALYTGNTNDFSQLQCKAVPVNTSDPLLVKWVKYDDN 203

* ***:***::**:* **:***: **:**: *::*:** * .:* *****::* **:.*

1-SST[*Helianthus tuberosus*]CAA08812 PVLLPPPGVGYKDFR**D**PSTLWSGPDGEYRMVMGSKHNETIGCALIYHTTNFTHFELKEEV 276

1-SST[*Cichorium intybus*]AFB83198.1 PILFPPPGVGYKDFR**D**PSTLWMGPDGEWRMVMGSKHNETIGCALVYRTTNFTHFELNEEV 288

1-SST[*Taraxacum koksaghyz*] PILFPPPGVGYKDFR**D**PSTLWRGPDGDWRMIMGSKHNETIGCALVYRTSNFTHFELSEEP 280

1-SST[*Taraxacum officinale*]CAB60153 PILFPPPGVGYKDFR**D**PSTLWRGPDGDWIMIMGSKHNQTIGCALVYRTSNFTHFELSEEP 280

1-FFT[*Helianthus tuberosus*]CAA08811 PILYTPPGIGLKDYR**D**PSTVWTGPDGKHRMIMGTKRGNT-GMVLVYYTTDYTNYELLDEP 269

1-FFT[*Taraxacum koksaghyz*] PILYIPPGIGPKDYR**D**PSTVWTGPDGKHRMIMGTKQNGT-GMVHVYHTTDFINYVLLDEP 262

1-FFT[*Cichorium intybus*]AFB83199.1 PILFTPPGIGLTDYR**D**PSTVWTGPDGKHRMIMGTKINRT-GLVLVYHTTDFTNYVMLDEP 262

*:* ***:* .*:*********:* ****. *:**:* . * * . :* *::: :: : :*

1-SST[*Helianthus tuberosus*]CAA08812 LHAVPHTGMW**E**CVDLYPVSTVHTNGLDMVDNGPNVKYVLKQSGDEDRHDWYAIGSYDIVN 336

1-SST[*Cichorium intybus*]AFB83198.1 LHAVPHTGMW**E**CVDLYPVSTTHTNGLEMKDNGPNVKYILKQSGDEDRHDWYAIGTFDPEK 348

1-SST[*Taraxacum koksaghyz*] LHAVPHTGMW**E**CVDLYPVSTTHTNGLDMMDNGPNVKYILKQSGDEDRHDWYAIGSFDPIN 340

1-SST[*Taraxacum officinale*]CAB60153 LHAVPHTGMW**E**CVDLYPVSTTHTNGLDMMDNGPNVKYILKQSGDEDRHDWYAIGSFDPIN 340

1-FFT[*Helianthus tuberosus*]CAA08811 LHSVPNTDMW**E**CVDFYPVSLTNDSALDMAAYGSGIKHVIKESWEGHGMDWYSIGTYDAIN 329

1-FFT[*Taraxacum koksaghyz*] LHSVPNTDMW**E**CVDFYPVSTINDSALDIAAYGSDIKHVIKESWEGHGMDLYSIGTYDAYK 322

1-FFT[*Cichorium intybus*]AFB83199.1 LHSVPDTDMW**E**CVDFYPVSTINDSALDIAAYGCDIKHVIKESWEGHGMDWYSIGTYDAMN 322

**:** *.**********:**** : ..*:: * .:*:::*:* : * *:**::* :

1-SST[*Helianthus tuberosus*]CAA08812 DKWYPDDPENDVGIGLRYDFGKFYASKTFYDQHKKRRVLWGYVGETDPQKYDLSKGWANI 396

1-SST[*Cichorium intybus*]AFB83198.1 DKWYPDDPENDVGIGLRYDYGKFYASKTFYDQHKKRRVLWGYVGETDPPKSDLLKGWANI 408

1-SST[*Taraxacum koksaghyz*] DKWYPDDPENDVGIGLRYDYGKFYASKTFYDQHKGRRVLWGYVGETDPPKDDLLKGWANM 400

1-SST[*Taraxacum officinale*]CAB60153 DKWYPDDPENDVGIGLRYDYGKFYASKTFYDQHKSRRVLWGYVGETDPPKDDLLKGWANM 400

1-FFT[*Helianthus tuberosus*]CAA08811 DKWTPDNPELDVGIGLRCDYGRFFASKSLYDPLKKRRITWGYVGESDSADQDLSRGWATV 389

1-FFT[*Taraxacum koksaghyz*] DKWTPDNPEFDVGIGLRVDYGRFFASKSLYDPLKKRRVTWGYVAESDSSDQDLNRGWATI 382

1-FFT[*Cichorium intybus*]AFB83199.1 DKWTPDNPELDVGIGLRVDYGRFFASKSLYDPLKKRRVTWGYVGESDSPVQDLNRGWATI 382

*** **:** ******* *:*.*:***::** * **: ****.*:*. ** .***.:

(b)

1-FEH1[*Cichorium intybus*]CAC19366 NWIN**D**PNGPMYFNGVYHLFYQYNPYGPLWG-NISWGHSISYDLVNWFLLEPALSPKEPYD 110

Invertase[*Cichorium intybus*]CAA72009 NWMN**D**PNGPMCYNGVYHLFYQYNPFGPLWNLRMYWAHSVSHDLINWIHLDLAFAPTEPFD 90

1-FEH[*Helianthus tuberosus*]AJW31156 NWMN**D**PNGPMLYQGVYHFFYQYNPLAPTFG-TIVWGHAVSHDLVNWIHLDPAIYPTHEPD 114

1-FEH[*Taraxacum koksaghyz*] NWMN**D**PNGPMLYKGVYHFFYQYNPYAATFGDLIIWAHAVSYDLVNWIHLDPAIYPTQEAD 115

1-FEHIIa[*Cichorium intybus*]CAC37922 NWMN**D**PNGPMLYQGVYHFFYQYNPYAATFGDVIIWGHAVSYDLVNWIHLDPAIYPTQEAD 115

1-FEHIIb[*Cichorium intybus*]AIP90173 NWMN**D**PNGPMLYQGVYHFFYQYNPYAATFGDVIIWAHAVSYDLVNWIHLDPAIYPTQEAD 115

**:*********** ::****:****** .. :. : *.*::*:**:**: *: *: *. *

1-FEH1[*Cichorium intybus*]CAC19366 INGCLSGSATILPGPRPIILYTGQDVNNSQVQNLAFPKNLSDPLLKEWIKWSGNPLLTPV 170

Invertase[*Cichorium intybus*]CAA72009 INGCLSGSATVLPGNKPIMLYTGIDTENRQVQNLAVPKDLSDPYLREWVKHTGNPIISLP 150

1-FEH[*Helianthus tuberosus*]AJW31156 ISSCWSGSATILPGNLPAMIYTGSDSTSRQVQDLAWPKNRSDPFLREWVKSTHNPIITPP 174

1-FEH[*Taraxacum koksaghyz*] IKSCWSGSATILPGNIPAMLYTGSDSKSRQVQDLAWPKNLSDPFLREWVKHPKNPLITPP 175

1-FEHIIa[*Cichorium intybus*]CAC37922 SKSCWSGSATILPGNIPAMLYTGSDSKSRQVQDLAWPKNLSDPFLREWVKHPKNPLITPP 175

1-FEHIIb[*Cichorium intybus*]AIP90173 SKSCWSGSATILPGNIPAMLYTGSDSKSRQVQDLAWPKNLSDPFLREWVKHPKNPLIIPP 175

..* *****:*** * ::*** * . ***:** **: *** *.**:* . **::

1-FEH1[*Cichorium intybus*]CAC19366 DDIKAGQFR**D**PSTAWMGPDGKWRIVIGSEIDGHGTALLYRSTNGTKWIRSKKPLHFSSKT 230

Invertase[*Cichorium intybus*]CAA72009 EEIQPDDFR**D**PTTTWLEEDGTWRLLVGSQKDKTGIAFLYHSGDFVNWTKSDSPLHKVSGT 210

1-FEH[*Helianthus tuberosus*]AJW31156 EGVKDDCFR**D**PSTAWLGPDGLWRIVVGGDRDNNGMAFLYQSPDFVTWTRYENPLAAADST 234

1-FEH[*Taraxacum koksaghyz*] EGVKDDCFR**D**PSTAWRGPDGVWRIVVGGDRDNNGMAFLYQSTDFVNWKRYDQPLSSAVAT 235

1-FEHIIa[*Cichorium intybus*]CAC37922 EGVKDDCFR**D**PSTAWLGPDGVWRIVVGGDRDNNGMAFLYQSTDFVNWKRYDQPLSSADAT 235

1-FEHIIb[*Cichorium intybus*]AIP90173 EGVKDDCFR**D**PSTAWRGPDGVWRIVVGGDRDNNGMALLYQSTDFVNWKRYDQPLSSAIAT 235

: :: . ********:*:* ** **:::*.: * * *:**.* : ..* . ..** *

1-FEH1[*Cichorium intybus*]CAC19366 GMW**E**CPDFYPVTNGDKKGLDTS-VQ-GNNTLHVLKVSFN--SREYYVIGTYDPIKDKFSV 286

Invertase[*Cichorium intybus*]CAA72009 GMW**E**CVDFFPVWVDSTNGVDTSIINPSNRVKHVLKLGIQDHGKDCYLIGKYSADKENY-V 269

1-FEH[*Helianthus tuberosus*]AJW31156 GTW**E**CPDFFPVQLNSTNGLDTSVVS-SGSVLHVMKAGFE--GRDWYTLGTYSPDRENF-L 290

1-FEH[*Taraxacum koksaghyz*] GTW**E**CPDFYPVPLNSTNGIDTS-VY-SGSVIHVMKAGFE--GHDWYTIGTYSSDRENF-L 290

1-FEHIIa[*Cichorium intybus*]CAC37922 GTW**E**CPDFYPVPLNSTNGLDTS-VY-GGSVRHVMKAGFE--GHDWYTIGTYSPDRENF-L 290

1-FEHIIb[*Cichorium intybus*]AIP90173 GTW**E**CPDFYPVPLNSTNGLDTS-VY-GGSVRHVMKAGFE--GHDWYTIGTYSPDRENF-L 290

* ******* **:** ...:*:*** : .. . **:* .:: ..: * :*.*.. .::: :

1-FEH1[*Cichorium intybus*]CAC19366 VTNDFMVSNT--QFQYDYGRYYASKSFYDSVNQRRVIWGWVNEGDSESDAVKKGWSGLQS 344

Invertase[*Cichorium intybus*]CAA72009 PEDELTLS----TLRLDYGMYYASKSFFDPVKNRRIMTAWVNESDSEADVIARGWSGVQS 325

1-FEH[*Helianthus tuberosus*]AJW31156 PQNGLSLSGSTLDLRYDYGNFYASKSFFDESKNRRVLWAWVPEKDSEEDDIEKGWAGLQT 350

1-FEH[*Taraxacum koksaghyz*] PQNGLSLTGSSLDLRYDYGQFYASKSFFDDAKNRRVLWAWVPETDSQADDIQKGWAGLQS 350

1-FEHIIa[*Cichorium intybus*]CAC37922 PQNGLSLTGSTLDLRYDYGQFYASKSFFDDAKNRRVLWAWVPETDSQADDIEKGWAGLQS 350

1-FEHIIb[*Cichorium intybus*]AIP90173 PQNGLSLTGSTLDLRYDYGQFYASKSFFDDAKNRRVLWAWVPETDAPEDDIEKGWAGLQS 350

: : :: :. *** :******:* ::**:: .** * *: * : .**:*:*:

**Figure S1. Partial multiple sequence alignment of several GH32-family proteins.** (a) Fructosyltransferases. (b) Fructan 1-exohydrolases and an invertase. Three GH32-family-specific conserved regions (x-x-x-**D**-P-D/N-G; R**D**P; and **E**C) including the catalytically active amino acids (bold) are marked with boxes. (a) The fructosyltransferase-specific motif x-A/G-Y/F (a) and the hydrolase-specific motif W-A/S/G-W (b) are shaded in gray.


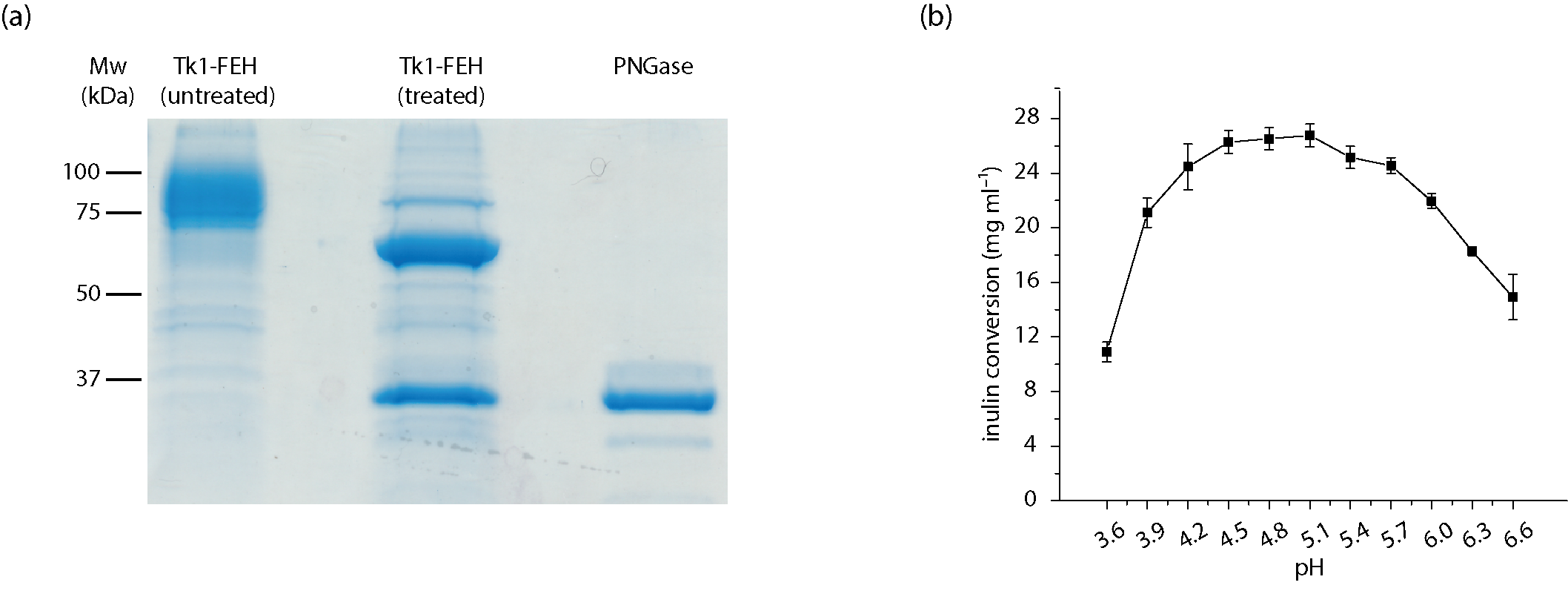


**Figure S2. Characterization of recombinant Tk1-FEH.** (a) Deglycosylated Tk1-FEH was visualized by SDS-PAGE using untreated Tk1-FEH and PNGase F alone as controls. (b) The pH-dependent conversion of inulin by recombinant Tk1-FEH was performed using 5% chicory inulin dissolved in 150 µl McIlvain buffer as a substrate. The effect of the pH value on inulin conversion was determined after 10 h at 30°C (n = 3; mean ± SD).


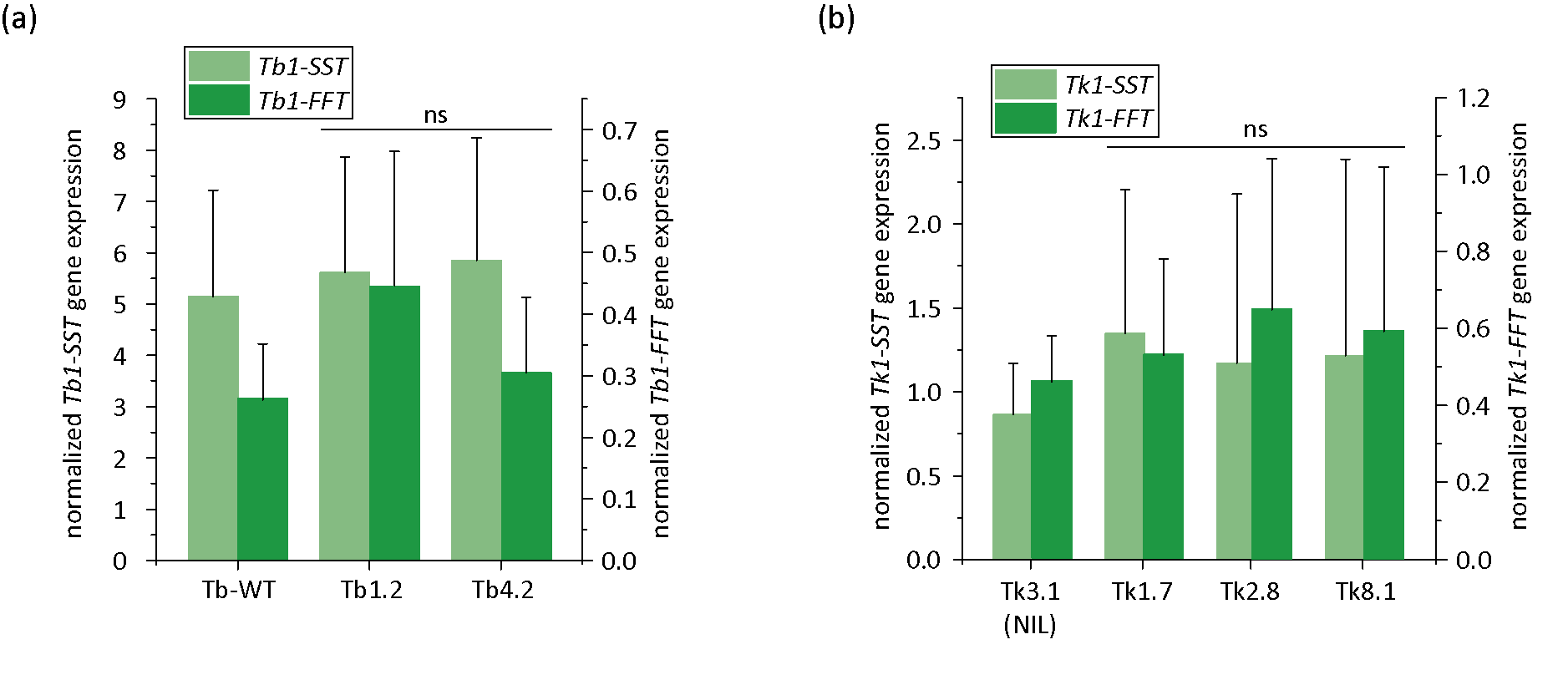


**Figure S3. *1-SST* and *1-FFT* gene expression levels in 18-week-old *T. brevicorniculatum* and *T. koksaghyz* plants overexpressing *Tk1-FEH*.** Quantitative determination of endogenous *1-SST* and *1-FFT* mRNA levels in roots of 18-week-old *T. brevicorniculatum* (a) and *T. koksaghyz* plants (b) overexpressing *Tk1-FEH*. Gene expression levels were normalized to the constitutive *TbEF1α* and *TkEF1α* genes, respectively. All values are means ± SD of 6–9 (*T. brevicorniculatum*) or 3–6 (*T. koksaghyz*) independently analyzed plants, each measured three times. Statistical significances (*P* ≤ 0.05) were determined by using the two-tailed Mann-Whitney test (ns = non-significant).

**Table S1. Analysis of Tk1-FEH expression in yeast cultures by mass spectrometry.** The Tk1-FEH-specific peptides listed below had a total coverage of 76.42%.

| Peptides | |
| --- | --- |
| NLIDHSIIESFGAGGK DPSTAWRGPDGVWR SNIDTTSFGAFVDIDPR RVLWAWVPETDSQADDIQK NPLITPPEGVKDDCFRDPSTAWR GPDGVWR NPLITPPEGVKDDCFR SCWSGSATILPGNIPAMLYTGSDSK MKAWSMK SEEISLR YDYGQFYASK VFQNQNGR ENFLPQNGLSLTGSSLDLRYDYGQFYASK VLWAWVPETDSQADDIQK TGYHFQPPSNWMNDPNGPMLYK LEDLKEAEVLDTNVIDPQALCAER ALWIDKSGK NIKDTIRLPNQK YSVLMCSDLSR SAEFVVDQTVK AGFEGHDWYTIGTYSSDRENFLPQNGLSLTGSSLDLR LEDLKEAEVLDTNVID IVVGGDRDNNGMAFLYQSTDFVNWK SGKQLIQWPVEEIEALR NLRPGSVLEIHGITASQADVTISFK NLSDPFLR AGFEGHDWYTIGTYSSDR NIKDTIR NLQEQTAIFFR  EAEVLDTNVIDPQALCAER | LNEVNLENK NLQEQTAIFFR EAEVLDTNVIDPQALCAER GALGPFGLLAMASK IVVGGDR NLIDHSIIESFGAGGKTCITSR SFFDDAKNR LNEVNLENK DNNGMAFLYQSTDFVNWK SNIDTTSFGAFVDID NPLITPPEGVK SNIDTTSFGAFVDIDPRSEEISLR GWAGLQSFPR DNNGMAFLYQSTDFVNWKR QLIQWPVEEIEALRLNEVNLENK FVQNEDAHLFVFNNGTQAIK VLWAWVPETDSQADDIQKGWAGLQSFPR LPNQKIEQPYR QVQDLAWPK QLIQWPVEEIEALR IVVGGDRDNNGMAFLYQSTDFVNWKR DTIRLPNQK ENFLPQNGLSLTGSSLDLR SRQVQDLAWPK SAEFVVDQTVKSTV DTIRLPNQKIEQPYR EWVKHPK |

**Table S2. Triterpene content of 40-week-old *T. brevicorniculatum* plants overexpressing *Tk1-FEH.*** Asterisks indicate statistically significant differences between the transgenic lines and their corresponding control (**P* ≤ 0.05 and ****P* ≤ 0.001; unpaired t-test).

| Plant lines | Total triterpenes ± SD  (mg g־¹ DW) | | Sterols ± SD  (mg g־¹ DW) | | Pentacyclic triterpenes ± SD  (mg g־¹ DW) | |
| --- | --- | --- | --- | --- | --- | --- |
| Tb-WT | 17.21 | (±3.44) | 4.19 | (±0.64) | 13.02 | (±2.81) |
| Tb1.2 | 35.26 | (±3.76)*** | 8.40 | (±0.60)*** | 26.85 | (±3.6)*** |
| Tb4.2 | 28.32 | (±8.93)* | 6.04 | (±1.43)* | 22.29 | (±7.58)* |

**Table S3. List of oligonucleotides used in this study.** Sequences are shown in the 5'→3' direction.

| Name | Sequence 5' →3' |
| --- | --- |
| 1-SST-SalI-fwd | AAAGTCGACATGGCTTCCTCAACCACC |
| 1-SST-NheI-rev | AAAGCTAGCTTAAGAACTCCACCCAGAAAG |
| 1-FFT-NotI-fwd | AAAGCGGCCGCATGAAAACCATCGAACCCTTTAGCGAC |
| 1-FFT-XbaI-rev | AAATCTAGATTAAAAAGGGTAAGCCTGAATTGACG |
| 1-FEH-GSP1-3‘ | ATAAAGTGGCTTATCATACAAACTCCAGTG |
| 3'-RACE-Adapter | GACTCGTGTGGACATCG |
| 1-FEH-GW1 | CCAGTTTACCAAATCTTGAGATACTGAATG |
| 1-FEH-GW2 | CAATCTCAGAAGTGTCAGGAACTCGAAC |
| 1-FEH-XhoI-fwd | AAACTCGAGATGAGCAAGCCTCTTTCCTCC |
| 1-FEH-XbaI-rev | AAATCTAGATTAAACTGTGCTTTTTACAGT |
| EF-1α-fwd | CGAGAGATTCGAGAAGGAAGC |
| EF-1α-rev | CTGTGCAGTAGTACTTGGTGG |
| 1-SST-RT-fwd | CGGTGGTGACATCACGAGTT |
| 1-SST-RT-rev | GGGAAAGGATCGAGTTGTGC |
| 1-FFT-RT-fwd | AAAACCGGAACCCATCTACTTCA |
| 1-FFT-RT-rev | GCCTATGTCGAGTGGAATGATTG |
| 1-FEH-RT-fwd | GCCTCTTTCCTCCTTTCTTGC |
| 1-FEH-RT-rev | CTTTTGATTGGGGAGCCGTAT |
| 1-FEH-fwd | AGCAAGCCTCTTTCCTCCTTTC |
| 1-FEH-KpnI-rev | AAAGGTACCTTAAACTGTGCTTTTTACAGT |
| 5'AOX | ACTGGTTCCAATTGACAAGC |
| CPT all-RT-fwd | TCGCCCTATCCCAGAACACATCGC |
| CPT all-RT-rev | ACCGACATGAGTGCTAGGAAGCCG |
| RTA-RT-fwd | CAGATGCAAATACAGAAAAACCGTTC |
| RTA-RT-rev | CCCATAGATCAGTAAAAGATCAGGAT |
| REF all-RT-fwd | GCAAGTGGTACAATGATACGG |
| REF all-RT-rev | CCATCATTCTTGGTCTTCACC |

**Table S4. Oligonucleotide efficiencies for qPCR.** Oligonucleotide efficiencies were determined by melt curve analysis at 60°C and / or 66°C.

|  | *T. brevicorniculatum* | *T. koksaghyz* |
| --- | --- | --- |
| Name of  primer pair | Primer efficiency  (%) | Primer efficiency  (%) |
| EF-1α | 101.0 (60°C) / 102.4 (66°C) | 97.2 (60°C) / 95.2 (66°C) |
| 1-SST-RT | 101.9 (60°C) | 97.9 (60°C) |
| 1-FFT-RT | 100.3 (60°C) | 95.1 (60°C) |
| 1-FEH-RT | 100.0 (60°C) | 102.9 (60°C) |
| CPT all-RT | 117.7 (66°C) | 121.3 (66°C) |
| RTA-RT | 106.5 (66°C) | 101.7 (66°C) |
| REF all-RT | 100.3 (66°C) | 101.7 (66°C) |

**Supplementary methods**

**Characterization of recombinant Tk1-FEH by mass spectrometry**

Recombinant Tk1-FEH was isolated by SDS-PAGE and the corresponding band was cut from the gel and washed three times with >10 volumes of Millipore water for 30 s while shaking. The gel slice was incubated with 300 µl 25 mM NH_4_HCO_3_ for 15 min while shaking, and the buffer was then replaced with 300 µl 25 mM NH_4_HCO_3_ in 50% acetonitrile and incubated for a further 15 min. The gel slice was then dehydrated in 100 µl acetonitrile for 5 min. Trypsin digestion was carried out using 10–20 µl modified trypsin (20 ng µl^-1^) in 10% acetonitrile / 25 mM NH_4_HCO_3_ on ice. After 90 min, the excess trypsin solution was replaced with 10% acetonitrile / 25 mM NH_4_HCO_3_ and the digestion was completed for 4–6 h at 37°C. The digested solution was transferred to a low-protein-binding tube and the trypsin was inactivated by adding 100 µl 50% acetonitrile containing 0.1% v/v formic acid and vortexing for 1 h. The solution was transferred into fresh low-protein-binding tubes and dried by vacuum centrifugation. The sample was reconstituted in 6 µl 5% acetonitrile containing 0.1% v/v formic acid and centrifuged (13,000 x g, room temperature, 5 min). Prior to analysis, 4 µl of the supernatant was transferred to an autosampler vial.

The peptides were separated using an Ultimate 3000 RSLCnano HPLC system (Dionex, Thermo Scientific, Darmstadt, Germany). The mobile phase for the loading pump was 0.05% (v/v) ultrapure water (A) and 80% acetonitrile / 0.05% (v/v) trifluoroacetic acid in ultrapure water (B). The sample (1 µl) was loaded on a trapping column (C18 PepMap 100, Thermo Fisher Scientific) and desalted for 5 min using eluent A at a flow rate of 20 µl min^-1^. The trap column was then switched online with the separation column (Acclaim PepMap100 C18, Thermo Fisher Scientific). The mobile phases for peptide elution comprised 0.1% (v/v) formic acid in ultrapure water (A*) and 80% acetonitrile / 0.08 % (v/v) formic acid in ultrapure water (B*). The peptides were eluted at a flow rate of 300 µl min^-1^ using the following gradient profile: 2.5 to 7.5% B* over 4 min, 7.5 to 40% B* over 28 min, 40 to 99% B* over 1 min, 99% B* for 10 min. The column was re-equilibrated with 99% A* for 24 min.

The HPLC system was coupled via a nanospray source to an LTQ Orbitrap XL mass spectrometer (Thermo Finnigan, Thermo Fisher Scientific). Full scans (*m/z* 350–1850) were acquired in positive ion mode by Fourier transform mass spectrometry (FT-MS) in the Orbitrap at a resolution of 70,000 (FWHM) with internal lock mass calibration on *m/z* 445.12003. An inclusion list with the precursor masses of the *in silico* digested proteins of interest was created using XCalibur Software v4.0.27.13. The 12 most intense ions from this list were fragmented in the linear ion trap by collision induced dissociation (28% normalized collision energy). Automatic gain control (AGC) was enabled with target values of 5 x 10^5^ and 1 x 10^5^ for full scans and MS/MS, respectively. One microscan was acquired per MS/MS spectrum and the maximum ion trap fill time was 100 ms ([Kalli and Hess, 2012](#_ENREF_24)). Dynamic exclusion was enabled with an exclusion duration of 30 s, a repeat count of 1, a list size of 500 and an exclusion mass width of ± 5 ppm. Unassigned charge states and charge =1 as well as ≥7 were rejected.

OMSSA v2.1.9 and X! Tandem v2013.09.01 were used for protein identification with a database comprising the target protein sequence and the reversed protein sequence as a decoy. A supplementary contamination database of human keratin and trypsin sequences was used with subsequent filtering of contamination hits. The parent mass error was set to 5 ppm and the fragment mass error was set to 50 ppm. Methionine oxidation was used as a variable modification and a maximum of two missed cleavages was tolerated. For OMSSA, a linear precursor charge dependency was set. For X! Tandem, noise suppression was enabled. A target/decoy approach was used for the statistical assessment of the peptide spectrum match (PSM) quality. PSM hits from OMSSA and X! Tandem were filtered (1) using a hit distinctiveness filter as described ([Specht et al., 2011](#_ENREF_48)), and (2) by removing all hits from peptides that occur as a target and as a decoy. These steps result in a maximum of one peptide hit per spectrum. Quality v2.02 was used for the statistical validation of protein identifications allowing only target hits with a q-value < 0.01. As an additional filtering step, all PSMs with a precursor mass deviation of more than 5 ppm were discarded.

**pH-dependent** **HPLC-coupled end point determination of 1-FEH characteristics**

The end point enzymatic assays were carried out as described in the section “HPLC-coupled end point determination of 1-FEH characteristics”. The optimal pH was determined by mixing the supernatant with 5% (w/v) chicory inulin dissolved in 150 µl McIlvain buffer (pH 3.6–6.6) and incubating for 10 h at 30°C shaking at 700 rpm.

**Statistical analysis**

The Shapiro-Wilk test ([Shapiro and Wilk, 1965](#_ENREF_47)) was used to check for the normal distribution of gene expression levels and metabolic data in Origin 2015 SR (OriginLab, Northampton, MA). Significant differences were tested using GraphPad Prism v3 (GraphPad Software, La Jolla, CA). Normally distributed datasets were analyzed using an unpaired two-tailed t-test. Datasets that did not show a normal distribution were analyzed using the two-tailed Mann-Whitney test. Correlations were analyzed using the Pearson r correlation or the Spearman rank correlation test depending on whether the data were normally distributed or not.
